# Study on Psychological Stress Perceived among Employees in an Italian University during Mandatory and Voluntary Remote Working during and after the COVID-19 Pandemic

**DOI:** 10.3390/ijerph21040403

**Published:** 2024-03-26

**Authors:** Loreta Tobia, Pierpaolo Vittorini, Giulia Di Battista, Simona D’Onofrio, Giada Mastrangeli, Pietro Di Benedetto, Leila Fabiani

**Affiliations:** 1 Department of Life, Health and Environmental Sciences, University of L’Aquila, 67100 L’Aquila, Italy; pierpaolo.vittorini@univaq.it (P.V.); giulia.dibattista1@graduate.univaq.it (G.D.B.); giada.mastrangeli@graduate.univaq.it (G.M.); pietro.dibenedetto@univaq.it (P.D.B.); leila.fabiani@univaq.it (L.F.); 2 Local Health Authority 01 Abruzzo, 67100 L’Aquila, Italy; drsimonadonofrio@gmail.com

**Keywords:** remote working, working from home, COVID-19, well-being, workplace, health promotion, university workers, GHQ-12

## Abstract

Objective of the Study: This cross-sectional study examined the perceived psychological well-being of administrative/technical employees and researchers/lecturers at the University of L’Aquila (Italy) during the COVID-19 pandemic. The study was carried out in two different periods of 2022: April 2022, when remote working was still mandatory, and December 2022, when the pandemic emergency had ended and, in Italy, remote working had become voluntary for two days a week and exclusively for administrative staff. Materials and Methods: Perceived psychological well-being was investigated using the GHQ-12 (Global Health Questionnaire, short-form with 12 items), a self-administered questionnaire created on Google Forms and sent via email to all the employees of the University of L’Aquila. Statistical analysis was conducted using means, standard deviations, and frequency tables for the descriptive analysis of socio-demographic data, while the *t*-test or Wilcoxon test and the Χ^2^ test were used to verify the statistical difference and association between categorical variables. Results: Overall, 365 employees, including 118 administrative/technical and 247 research/teaching staff, participated in the survey in April 2022 when remote working was mandatory. Among them, 219 (52.8%) were female and 196 (47.2%) were male. In December 2022, 266 employees engaged in voluntary remote working, including 184 (69.2%) women and 82 (30.8%) men, took part in the study. The most represented age group was 50–59 years old (36.3% of study participants). During mandatory remote working, 83.4% of lecturers reported a perceived level of psychological distress ranging from moderate to severe versus 69.5% of technicians. The percentage of self-reported psychological distress was higher among the technicians forced to work from home (n. 118–42.9%) vs. the technicians working from home on a voluntary basis (n. 157–57.1%), with GHQ score being >14 in 65.5% of enforced remote workers vs. 62.3% of voluntary remote workers. During mandatory remote working, there was a significant difference in the GHQ-12 score between administrative and research staff, particularly related to items such as loss of self-confidence, emotional pressures, and diminished productivity. Moreover, from the comparison between the group of administrative staff engaged in mandatory remote working and those in voluntary remote working for specific GHQ-12 items, a statistically significant difference emerged concerning the perception of not being able to overcome difficulties; the GHQ-12 score was higher in the first group. Significant differences in the overall GHQ-12 score were evident between male and female lecturers, as the latter reported higher levels of perceived stress during mandatory remote working. Discussion: The results confirm that remote working could be associated with a better psychological state of administrative university staff, especially in the case of voluntary remote working. During mandatory remote working, a difference was observed between teaching and administrative staff, with higher stress in the first group and among women. Therefore, our sample appears fragmented in the self-assessment of psychological well-being during remote working, possibly due to profound differences in the organization of work activities between lecturers and administrative employees. Additionally, the increased perception of stress by female lecturers compared to males may reflect gender disparities, as women working from home during the pandemic experienced an increased workload including domestic activities. Conclusions: Remote working is a type of working that has both advantages and disadvantages. An advantage is undoubtedly a better work–life balance; however, the risks of technostress, workaholism, increased sedentary behaviour, and social isolation are negative aspects. This study provides an indicative overview of the psychological state related to remote working in a university setting during the SARS-CoV-2 pandemic. The study might therefore serve as a starting point for further research on the impact of remote working on self-reported psychological well-being, especially in the university environment.

## 1. Introduction

Telecommuting, remote work, teleworking, and flexible work have been used in the literature as synonyms [1,2]. These different terms make it difficult to evaluate prior research and related findings. Therefore, to give a comprehensive definition and to discuss the related work, we rely upon the definition found in [2]: remote work “[…] is a work practice that involves members of an organisation substituting a portion of their typical work hours (ranging from a few hours a week to nearly full-time) to work away from a central workplace—typically principally from home—using technology to interact with others as needed to conduct work tasks. […]”. Remote working was significantly used worldwide during the 2019 SARS-CoV-2 pandemic. In 2017, in Italy, the percentage of “remote” workers was around 8%, the lowest value among all European Union countries [3]. During the COVID pandemic, the percentage of remote workers in Italy increased to 69%, and it is estimated that 81% of workers worldwide have changed their work settings [4]. *Remote working* is defined by Italian law 81/2017 as a flexible and simplified mode of work aimed at facilitating the reconciliation of life and work schedules and increasing productivity [5]. Numerous pre-pandemic literature studies report experiences of *remote working*. Evidence during the pandemic is less extensive, and even fewer studies investigate the university sector. In summary, there is no unanimous consensus regarding the advantages and disadvantages of remote working. The most common advantages are reduced commuting times, increased staff motivation and productivity, and better ability to meet deadlines. On the other hand, the most frequently highlighted disadvantages include difficulty in monitoring performance and communication problems among colleagues, the absence of ergonomic devices in the domestic environment, and an increased possibility of musculoskeletal symptoms [6,7,8,9,10,11,12,13,14,15,16,17,18,19,20,21]. The related work section contains a detailed discussion of the available evidence.

Since March 2020, to safeguard the health of students and employees and to contain SARS-CoV-2 infection, the University of L’Aquila adopted a series of measures, including the introduction of distance learning for all degree programmes and remote working for almost all its employees. Further decree-laws implemented provisions on remote working during the public health emergency. The Italian Legislative Decree No. 24 of 24 March 2022 lifted some restrictions and ended the state of emergency. Subsequent decrees have extended vulnerable workers’ right to remote work (currently until 31 December 2023). After the official end of the COVID-19 public health emergency, the University of L’Aquila extended the right to work remotely to administrative and technical staff for a maximum of two days per week. Workers’ psychological well-being and stress levels have been carefully evaluated during the pandemic period using the GHQ-12 questionnaire [22]. The GHQ-12 is a valuable tool for detecting psychological distress, which has been used in several studies that investigated workers’ psychological well-being during the COVID-19 pandemic [23,24,25,26]. In this study, we report the impact of remote working on self-reported productivity and how it influenced the personal and professional well-being of employees at the University of L’Aquila using the GHQ-12 questionnaire. 

**Related work:** To identify the related work, we searched the main digital libraries (e.g., PubMed), without limiting the publication dates, using the following keywords: “remote work”, “telework”, “psychological well-being”, “employees” “University workers”, “COVID-19 Pandemic”, “GHQ”, and “something else”. Therefore, we filtered the retrieved references in terms of relevance.

Finally, we structured the related work by examining the pros and cons of remote work, then we summarized the main findings regarding the effects of remote work during the recent COVID-19 outbreak. The possible impact of remote work—in terms of both advantages and disadvantages—has long been studied. In a recent survey on employees by Eurofound and the International Labour Office [4], workers reported that reduced commuting time and increased autonomy lead to better work–life balance (mainly women) and higher productivity. At the same time, workers mentioned a tendency for longer working hours and an increased overlap between work and personal life. A literature review focusing on factors that could affect individual and organizational working outcomes [18] showed:

(A) As positive outcomes, higher self-reported productivity and the possibility of taking care of family members;
(B) As adverse outcomes, reduced communication with co-workers and the need for an explicit supervisor’s trust and support.

A study focusing on managers’ viewpoints [27] showed several concerns, i.e., the lack of face-to-face communication and the associated benefits, reduced overall team effectiveness, and the difficulty of managing and monitoring remote workers’ performance. Similar worries are reported in [28], especially the uncertain advantages coupled with the immediate disadvantages. A recent literature review selected seven articles on the psychological impact of remote work on employees [14]. The main findings are that remote work can help workers’ engagement and connectivity among staff, even if it can reduce the work–home boundary and increase both fatigue and mental demand. A further study [15] found that the possibility of working flexibly yields several adverse impacts on well-being due to overworking and reduced time for recuperation. In [16], a cross-national survey confirmed the pros and cons mentioned above, but it highlighted a reduced overall satisfaction with life in the partners of remote workers. The study in [19] reported that a separate room for remote work ameliorated spatial but not mental overlap of work and private life. Moreover, remote work facilitated restoration, conditional on gender: women reported less effective restoration, while men reported more effective restoration. According to [29], 86.2% of female teachers perceived an increased workload while working from home, and indicators of depression were more prevalent among female teachers compared to males, who exhibited a more significant lack of desire and motivation. The authors of [30] reported worse results: stress is experienced when private activities occur in the work sphere (and vice versa), but spatial and temporal boundaries do not prevent it. A systematic literature review [31] summarized the results from sixty-three studies. It concluded that there is a positive relationship between remote work and well-being (positive emotions, increased job satisfaction and organizational commitment, ameliorated emotional exhaustion), even if social isolation was the main drawback. A study on journalists [20] reported that remote work caused less stress but increased negative emotions such as loneliness, irritation, worry, and guilt. Furthermore, remote workers experienced more mental illness than office workers. The specific type of work, the investigation and the limited sample may have caused these countertrending results.

Table 1 lists the references for all papers; in case of a review, the main pros and cons are reported, including a summary of the most common advantages and disadvantages of remote work.

If, on the one hand, the impact of remote working has been largely studied, on the other hand, the role of remote working during the COVID-19 outbreak is a recent field of study. Hereafter, we review the related papers by first summarizing the general role of remote work during the COVID-19 pandemic, then discussing its impact, and finally by focusing on the university sector, which is also the focus of our research. According to the Eurofound EU Agency [4], the experience of working from home during the COVID-19 crisis appears to have been positive for many employees. The preferred type of remote work was several times a week, with very few respondents indicating they would like to work remotely daily. Not surprisingly, respondents working in the health sector more often reported being emotionally drained, as also highlighted in [25]. The cross-sectional study in [21] reported that a significant portion of workers were less stressed and equally satisfied than when working at the office. Conversely, workers reported being less productive, and that neck pain worsened. A further study [32] stated that the psychological well-being of employees was not affected by remote work. Moreover, the study identified several stress-related factors, i.e., role ambiguity, organizational climate, and job dissatisfaction. A survey conducted in Lithuania [33] identified the characteristics of the most satisfied and dissatisfied remote workers: (i) most satisfied: millennial women with a higher education degree, 4–10 years of professional experience, working from home two days a week, management, and administration fields; (ii) most dissatisfied: men close to retirement, with a university degree, 20 years or more of professional experience, and experience with remote work only during the pandemic. Another survey, though conducted in France [34], revealed the superiority of the influence of crisis-specific variables (over the non-crisis-specific ones), especially stress, together with isolation and high work interdependence. An Italian survey [24] found that remote workers reported isolation, lack of support, stress, and overwork. At the same time, the authors noticed the rise of unhealthy behaviours (e.g., smoking, alcohol abuse, eating) among the respondents who reported higher levels of psychological distress. In [35], the authors identified both work overload and stress in remote workers, and thus suggested that job crafting could play a crucial role as a protective factor. A study [36] on the relationship between workaholism and technostress pointed out that excessive and compulsive work was a significant predictor of technostress, particularly during the COVID-19 crisis. Employees’ mental health conditions under COVID-19 restrictions were reviewed in [37]. The authors highlighted that the capability of practicing social distancing positively affected employees’ mental health. On the contrary, lockdown, quarantine, and resuming work caused mild to severe mental health issues. Finally, the work discussed in [38] reports the decreased overall physical and mental well-being of office workstation users, associated with several factors like reduced physical exercise, comfort food intake, and work distractions (e.g., children at home). There are very few specific studies on the well-being of university workers during COVID-19. In [39], the authors enrolled university staff to investigate the role of leaders during the crisis. The results showed that high/low authoritarian leadership had an enhancing/protective effect on technostress, respectively. A study closely related to our research was recently conducted at a Chilean university [40]. It revealed a higher percentage of stress which was present in 55.7% of the employees, associated with depression in 26% of them and anxiety in 29.2%. The highest rates of stress were observed in women, academics, those under 40 years of age, and in contract workers.

Table 2 lists the findings mentioned above and summarises the most frequent results.

Finally, the GHQ-12 has been used in several studies to detect psychological distress. For example, Italian research conducted during the pandemic found that community pharmacists reported a moderate stress level [25], probably due to their direct and continuous contact with the population, and their consequently high risk of infection. One of our previous studies focused on the well-being of university staff during the pandemic in 2021 when remote working was mandatory, showing that lecturers perceived an increased workload compared to administrative staff, with percentages of 73.6% and 60%, respectively. According to this research, “the administrative staff group provided the most positive overall rating (very satisfactory or satisfactory) with higher percentages than the teaching staff (*p* < 0.001), with a trend that was even more evident in women” [23]. Another study assessed the physical and psychological health of construction workers and the relationship between their well-being and the preventive measures applied against SARS-CoV-2 using the GHQ-12 questionnaire. The results showed enhanced well-being due to the preventive measures adopted [26].

## 2. Materials and Methods

In Italy, remote work is divided into two typologies: remote working and teleworking. The Legislative Decree n. 81 of 22 May 2017, and amendments thereof, define remote working as the possibility of working anywhere, with any technology device and with time flexibility. The EU Framework Agreement of 16 July 2022 defines telework as a modality where the working hours are strictly defined, and the workers must stay home to perform their jobs. In our study, we focused on remote working organized as follows: university employees worked at home using their own resources (i.e., computer, network connection) and—when remote working became voluntary in December 2022—only for two days a week. We describe the effects of remote working on the perceived health between:

(1) Two different occupations (i.e., administrative/technical vs. research/teaching) during April 2022 (when remote working was mandatory);
(2) Two different types of remote working (i.e., mandatory vs. voluntary) during the two different periods of April 2022 and December 2022 (only for administrative/technical staff). To collect the data, we administered an online questionnaire to the employees of the University of L’Aquila.

To collect data to support our objectives, we developed a questionnaire structured into several sections and we emailed it to the employees of the University of L’Aquila. Participation was free and voluntary. The first section contained general socio-demographic and occupational information. The second section included questions about specific health aspects (e.g., psychological health, anxiety, demoralization). All these aspects were investigated with categorical answers (i.e., worsening/unchanged/improvement). The third section was the General Health Questionnaire Short Form 12 (GHQ-12), a 12-item self-reporting instrument for detecting mental disorders in the community and in non-psychiatric clinical settings [22]. This questionnaire asks respondents to report how they have been feeling over the past few weeks using a 4-point scale (“more than usual, as usual, less than usual, and much less than usual”), and it is scored using a numerical response (Likert scale: 0–1–2–3), resulting in a scale ranging from 0 to 36. A score below 15 indicates a normal stress level, a score between 15 and 20 shows the presence of stress, and a score above 20 indicates severe psychological distress [40]. In our results, they were, respectively, represented as “Typical”, “Evidence of distress” and “Severe problem”. The fourth section of the questionnaire asked for general opinions about remote working. The whole questionnaire is reported in the Supplementary Materials. The questionnaire was administered to administrative/technical and research/teaching staff in April 2022 (when remote working was mandatory) and only to administrative/technical staff in December 2022 (when remote working was voluntary). For the purposes of this study, we focused on analysing the GHQ-12 and its relationship with the different occupations (i.e., administrative/technical or research/teaching staff) and the two different periods (i.e., April 2022 vs. December 2022), i.e., two different types of remote work, mandatory and voluntary. We used means, standard deviations, and frequency tables as descriptive statistics; *t*-test (or Wilcoxon test in case of a non-normal distribution or categorical data) and χ2 test to test the statistical difference and associations. Note that the anonymity of the participants prevented us from performing paired tests. The analyses were performed using R4.2.1. Differences/associations were considered significant for a *p*-value < 0.05, whereas we also reported differences with a *p*-value < 0.10. The study was authorized by the Internal Review Board of the University of L’Aquila (IRB: n. 31/2020).

## 3. Results

We invited 878 workers to participate in the study in April 2022, when remote working was mandatory, three months after the fourth Italian lockdown period, and 365 answered the questionnaire (41.6%). We invited 171 workers to participate in December 2022, four months after elective remote working was established (i.e., in September 2022), and 157 of them answered the questionnaire (91.2%). Note that we sent out the questionnaire only to administrative/technical staff, as they were the only ones still allowed to work remotely. Moreover, the administrative/technical staff in April and December were the same cohort. Finally, elective remote working was characterised by a maximum of 2 days per week in remote working.

Table 3 outlines the descriptive characteristics of the sample. A total of 365 employees participated in the survey in April 2022 (highlighted with the caption “Mandatory remote working—different occupation”) and 275 employees participated in April/December 2022 (reported as “Administrative/technical staff—mandatory vs. voluntary remote working”).

In April 2022, 247 lecturers/researchers and 118 administrative employees participated in the study by responding to the questionnaire, totalling 365 university employees forced to work from home. Among them, 219 (52.8%) were female and 196 (47.2%) were male. The most represented age group was 50–59 years old (36.3% of study participants). Most workers took less than 30 min to commute to their workplace and primarily resided in the municipality of L’Aquila.

In the survey conducted in December 2022, 60–70% of respondents found the conditions of working from home and the computer equipment provided by the University to be satisfactory; 37% of employees reported an improvement in their night-time sleep quality and duration. There was an increase in the consumption of fruits and vegetables, along with a reduction in comfort food. Physical activity and weight remained unchanged, while levels of concentration increased. Relationships with colleagues improved, and work–life balance was more harmonized; 44.5% of respondents reported an improvement in their psychophysical health, despite an increase in symptoms of anxiety and musculoskeletal disorders. Overall, 80.1% found the experience of remote working satisfying and were content with the option of combining in-person and remote work in their employment contract.

Among the respondents, 244 (58.7% of the entire sample from April 2022) reported evident psychological distress. In December 2022, 157 administrative employees engaged in voluntary remote working participated in the study. Table 4 divides the distress scores by job position and type of remote working. The numbers are expressed in absolute frequencies, percentages, and respective *p*-values. The results show a significant difference in GHQ-12 (psychological distress) between technical and teaching staff in April. Furthermore, in December 2022, when the possibility of working remotely on a voluntary basis was only given to administrative staff, there was a reduction in severe distress, decreasing from 17% to 8.9% (Table 4), although the difference was not statistically significant.

Table 5 contains a detailed analysis of the GHQ-12. It reports the average values (with standard deviation) of the GHQ-12 total score and of each item, for the two different occupations and the two types of remote working, with the corresponding *p*-value. The (+) mark indicates that a higher value is associated with higher psychological distress. The (−) mark means the opposite. It also presents an analysis of the total GHQ-12 scores and item-wise scores within the same group of workers (administrative staff) between April and December, along with the corresponding *p*-values. Statistically significant differences emerge in the total GHQ-12 score between technical and research staff. Specifically, the teaching staff exhibited a higher total GHQ-12 score compared to the administrative staff, indicating poorer mental health. They also reported a greater loss of confidence while telecommuting compared to the administrative group (*p* = 0.019).

When evaluating the GHQ-12 scores within the same group of workers who chose remote working versus those who were enforced in April 2022, the former group shows an overall lower GHQ-12 score compared to the latter, though the differences are not statistically significant. Further analysis of the two groups (teaching and administrative staff) based on individual GHQ-12 scores reveals statistically significant differences in the variables “Playing a useful part” (+), “Felt constantly under strain” (−), and “Losing confidence” (−) in April, indicating a more challenging situation for teaching staff. In the analysis conducted in December, there is an improvement in the GHQ-12 scores, although not statistically significant. Statistically significant differences are observed in the variables “Couldn’t overcome difficulties” and “feeling unhappy and depressed” (−).

Table 6 summarizes the results of the answers given to the additional questions regarding general psychological health, anxiety, and demoralization, expressed in terms of three categories (worsening, unchanged, and improvement). The numbers represent the absolute frequencies; the relative frequencies calculated by column and the respective *p*-value are reported in parentheses.

There is a statistically significant difference in the perceived psychological health in the sample from April 2022, with a decline noted in the group of researchers. Conversely, no differences are observed between the two groups in terms of perceived anxiety and demoralization. 

We then examined the data collected in December from a homogeneous group of employees. The analysis of the variables of perceived psychological health, anxiety, and demoralization between those who chose and those who were forced to work remotely shows a statistically significant difference in the improvement of perceived psychological health, with 44% of workers in voluntary remote working reporting improvement vs. 30% of enforced remote workers.

Table 7 shows that there was no statistically significant difference in the GHQ scores between males and females in the groups of lecturers and technicians enforced to work remotely. However, a difference is observed between males and females in the GHQ scores for the group of technicians who were enforced to work remotely, thus indicating a stronger deterioration in the perceived psychological health among female employees.

Considering the overall GHQ-12 score, Table 8 shows a significant difference between males and females exclusively within the teaching staff who engaged in remote working in April, with higher scores for women for the following items: capacity to make decisions, feeling constantly under strain, and feeling reasonably happy. There are no statistically significant differences between males and females in the other two groups, in April and December, for both the total GHQ-12 score.

## 4. Discussion

The discussion is structured in terms of the different objectives (i.e., perceived health quality in (i) the two occupations and (ii) in the administrative staff during mandatory and voluntary remote working), along with a specific discussion regarding gender differences.

**Different occupations**. Our results (Table 3) highlight that during the COVID-19 pandemic, most university staff showed evidence of distress (58.7%), and severe problems were present in a non-negligible proportion (19.4%). Researchers/lecturers reported worse health than the administrative/technical staff (Table 5) and a worsening in their psychological health, anxiety, and demoralization (Table 6). According to the specific items of the GHQ-12, teaching staff experienced a more substantial loss of confidence, the feeling of not playing a helpful role, and a more remarkable inability to overcome difficulties when compared to administrative personnel. These results may be explained because remote work was introduced mandatorily and entirely without prior experimentation [39]. In particular, the perception of distress among lecturers in our university could have been amplified by two-fold factors. First, e-learning systems were adopted without technical and organizational support for their work activities and for the first time in their professional careers. Second, the role of responsibility felt towards their organization during the emergency in domestic isolation [39,40,41]. Recent findings support this view: a Finnish study noted that the sudden shift to remote teaching, as well as teaching during the COVID-19 pandemic, may have caused some changes in the demands teachers faced in their work. Moreover, UNESCO reported that not all teachers were provided with support during the sudden changes, nor with the requirements that the situation created for the teachers [42,43]. Finally, even if few studies have investigated the psychological state of university teaching/research staff in remote working during the COVID-19 pandemic, our results align with the findings of De Sio et al. [24]. In contrast, the Gallardo et al. study [40] reports worse psychological conditions.

**Administrative staff during mandatory and voluntary remote working**. Concerning the perceived psychological distress among employees during the remote work experience, a slight difference emerged, albeit not statistically significant, between mandatory and voluntary remote working. The worse state of well-being found during mandatory remote working may be linked to the fact that in the second phase of the study, remote working, in addition to being voluntary, was also partial (allowing for a maximum of 2 days per week). Moreover, working remotely only a few days a week could lead to lower stress and work overload. Similarly to our results, the study on administrative employees in Lithuania showed that younger women with a higher level of education and working two days a week were more satisfied with remote working than men close to retirement [33]. Other studies found that remote work did not affect psychological well-being, though this could be influenced by occupational stress, role ambiguity, organizational climate, and job satisfaction [32].

**Gender differences.** From the results, a difference in the perceived psychological health between men and women also emerges, highlighting a higher level of distress in female teachers compared to males during mandatory remote work. In fact, female teachers showed a greater sense of feeling under pressure than men. Our results were supported by Zapata et al. [29]. On the contrary, we did not observe differences in perceived stress between genders within the administrative staff engaged in remote working during voluntary remote work. Some authors report a higher degree of distress in female workers, noting their increased involvement in household responsibilities, particularly during the domestic isolation associated with the pandemic [29,43,44,45]. Similarly, other studies indicate greater apprehension, organizational difficulties, and family challenges for women engaging in telecommuting [38,40]. Finally, a recent review revealed a higher level of technostress in female workers than males [46], possibly justified by an imbalanced use of technology in the workplace [2].

**Limitations of the study.** The present study has some limitations. First, it examines a limited sample size that may not be representative of the general population. Additionally, data are self-reported and therefore subjective, thus suggesting the possibility of response bias.

This study did not allow for an investigation into the mental and psychological health of remote workers under normal circumstances, as the COVID-19 pandemic has affected the overall well-being of the population.

A further limitation is the lack of consideration of individual factors (family circumstances, physical health, economic condition, etc.) that may have influenced the psychological state of the studied group.

## 5. Conclusions

This paper summarised the main findings related to the psychological stress suffered by the employees of the University of L’Aquila in remote working in two different periods and comparing research/teaching vs. administrative/technical staff. The results show that most university staff had evidence of distress, severe problems were not negligible, and researchers/teachers reported worse psychological conditions than the administrative/technical staff. When comparing mandatory vs. elective remote working, the GHQ-12 did not highlight changes, even if employees reported improved psychological health during elective remote working. Finally, female researchers/teachers had worse psychological conditions, compared to males, during mandatory remote work.

Among the positive effects of the 2020 lockdown, we cannot overlook the contribution and impetus towards an increase in remote work. This approach proved crucial during critical phases, allowing the university to continue providing its services. Currently, it continues to receive positive feedback and approval from the staff. It is hoped that this work modality can be further applied in the future, considering any existing challenges. It is worth noting that remote working did not lead to a reduction in productivity, coupled with high levels of satisfaction among both workers and the organization. Remote work has had a positive effect in terms of stress reduction, especially for university administrators. However, challenges related to remote working persist, such as the risk of technostress, workaholism, and social isolation. It is important to emphasize that the pandemic may have influenced workers’ psychological condition, regardless of the type of work arrangement. We hope to conduct further research to investigate the effects of remote working on mental health, specifically for certain occupational categories.

## Figures and Tables

**Table 1:** List of the analysed papers and a summary regarding the most frequent advantages and disadvantages of remote work (The sign ✓ stands for review).

Paper	Review	Advantages	Disadvantages	Sample Size (M/F)
*General results*				
[2]	✓	reduced commuting time, increased autonomy, better work–life balance (in particular, for women), higher productivity	longer working hours, increased overlap between work and personal life, reduced well-being outcomes for “high-mobile” workers	
[16]	✓	higher self-reported productivity, possibility of taking care of family members	reduced communication with co-workers, the need for an explicit supervisor’s trust and support	217 (135/82)
[30]			lack of face-to-face communication and the associated benefits, reduced overall team effectiveness, difficulty of managing and monitoring remote workers’ performance	26
[31]	✓		uncertain benefits of remote work	37,533
*Results related to health*				
[12]		increased worker engagement and connectivity among staff	blurred work–home boundary, increased fatigue and mental demand	20 (7/13)
[13]			overworking and reduced time for recuperation	
[14]			reduced overall satisfaction with life in partners of remote workers	2431
[17]		separate room for remote work ameliorated spatial overlap of work and private life, facilitated restoration	separate room for remote work did not ameliorate mental overlap of work and private life	18 (0/18)
[27]			stress when private activities occur in the work sphere, spatial and temporal boundaries do not prevent it	-
[23]		positive relationship between remote work and well-being, namely: positive emotions, increased job satisfaction and organisational commitment, ameliorated emotional exhaustion	social isolation	510 (245/265)
[18]		less stress	negative emotions such as loneliness, irritation, worry, and guilt, more mental illness	128 (56/72)
*Summary*				
		better work–life balance, higher productivity, facilitated restoration, positive emotions	blurred private/work boundary, increased mental demand, social isolation	

**Table 2:** List of the papers analysed and a summary of the main findings.

Paper	Findings
*Role of remote working during COVID-19 crisis*	
[41]	positive experience, remote working several times a week, workers of the health sector emotionally drained
*Impact of remote working during COVID-19 crisis*	
[38]	emotionally exhausted health care workers
[19]	less stressed, equally satisfied, less productive, neck pain worsened
[20]	unaffected psychological well-being; role ambiguity, organisation climate and job dissatisfaction as stressing factors
[21]	profile of most satisfied/dissatisfied remote workers
[32]	superior influence of crisis-specific variables over the non-crisis-specific ones
[33]	workers reported isolation, lack of support, stress, and overwork; rise of unhealthy behaviours
[42]	work overload and stress, suggested job crafting as a protective factor
[29]	excessive and compulsive work was a significant predictor of technostress
[43]	social distancing was positively related to employees’ mental health; lockdown, quarantine, and resuming work could cause mild to severe mental health issues
*Impact of COVID-19 crisis on university staff*	
[44]	high/low authoritarian leadership had an enhancing/protective effect on technostress
[36]	stress (in particular for women, academic staff, and contract workers), depression, anxiety, and burnout
*Summary*	
	positive experience, decreased mental well-being, increased stress (with one exception), rise of unhealthy behaviours

**Table 3:** Descriptive characteristics of the sample.

Characteristics	Categories	n	%
***MANDATORY REMOTE WORKING—DIFFERENT OCCUPATIONS***			
**Occupation**	Administrative/technical	118	32.3%
	Research/teaching	247	67.7%
**Sex**	Female	219	52.8%
	Male	196	47.2%
**Age range**	<30	37	8.9%
	30–39	53	12.7%
	40–49	88	21.2%
	50–59	151	36.3%
	≥60	87	20.9%
**GHQ**	Typical	91	21.9%
	Evidence of distress	244	58.7%
	Severe problem	81	19.4%
***ADMINISTRATIVE/TECHNICAL STAFF—MANDATORY* vs. *VOLUNTARY REMOTE WORKING***			
**Month**	April	118	42.9%
	December	157	57.1%
**Sex**	Female	184	69.2%
	Male	82	30.8%
**Age range**	<30	1	0.4%
	30–39	9	3.3%
	40–49	53	19.8%
	50–59	158	59%
	≥60	47	17.5%
**GHQ**	Typical	91	34.5%
	Evidence of distress	140	53%
	Severe problem	33	12.5%

**Table 4:** The distribution of distress between occupations and the type of remote working. The numbers are the absolute frequencies, the percentages (calculated by column), and the corresponding *p*-value (n.s. stands for not significant).

GHQ	Administrative/ Technical	Research/ Teaching	*p*-Value
**Typical**	36 (30.5%)	41 (16.6%)	0.009
**Evidence of distress**	62 (52.5%)	150 (60.7%)	
**Severe problem**	20 (17.0%)	56 (22.7%)	
**GHQ**	**Mandatory** **remote working**	**Voluntary** **remote working**	***p*-value**
**Typical**	36 (30.5%)	55 (37.7%)	n.s.
**Evidence of distress**	62 (52.5%)	78 (53.4%)	
**Severe problem**	20 (17.0%)	13 (8.9%)	

**Table 5:** GHQ-12 average values (with standard deviation) between occupations and types of remote workin. (n.s. stands for not significant).

Items	Administrative/ Technical	Research/ Teaching	*p*-Value
**GHQ total score (+)**	15.9 (4.7)	17.2 (4.5)	0.008
**Able to concentrate (+)**	1.07 (0.4)	1.19 (0.6)	0.068
**Loss of sleep over worry (−)**	1.58 (0.8)	1.74 (0.8)	0.068
**Playing a useful part (+)**	0.98 (0.5)	1.14 (0.6)	0.031
**Capable of making decisions (+)**	1.02 (0.4)	1.03 (0.4)	n.s.
**Felt constantly under strain (−)**	1.97 (0.7)	2.14 (0.7)	0.034
**Couldn’t overcome difficulties (−)**	1.90 (0.6)	1.92 (0.7)	n.s.
**Able to enjoy day-to-day activities (+)**	1.09 (0.9)	1.27 (0.8)	0.050
**Able to face problems (+)**	1.04 (0.6)	1.07 (0.5)	n.s.
**Feeling unhappy and depressed (−)**	1.76 (0.7)	1.85 (0.8)	n.s.
**Losing confidence (−)**	1.63 (0.8)	1.83 (0.8)	0.019
**Thinking of self as worthless (+)**	1.35 (0.8)	1.20 (0.8)	0.072
**Feeling reasonably happy (+)**	1.06 (0.7)	1.17 (0.6)	0.062
**Items**	**Mandatory** **remote working**	**Voluntary** **remote working**	***p*** **-value**
**GHQ total score (+)**	15.9 (4.7)	15.2 (4.3)	n.s.
**Able to concentrate (+)**	1.07 (0.4)	1.00 (0.5)	n.s.
**Loss of sleep over worry (−)**	1.58 (0.8)	1.52 (0.8)	n.s.
**Playing a useful part (+)**	0.98 (0.5)	0.90 (0.5)	n.s.
**Capable of making decisions (+)**	1.02 (0.4)	0.96 (0.4)	n.s.
**Felt constantly under strain (−)**	1.97 (0.7)	1.79 (0.8)	0.083
**Couldn’t overcome difficulties (−)**	1.90 (0.6)	1.57 (0.8)	0.001
**Able to enjoy day-to-day activities (+)**	1.09 (0.9)	1.03 (0.8)	n.s.
**Able to face problems (+)**	1.04 (0.6)	0.96 (0.5)	n.s.
**Feeling unhappy and depressed (−)**	1.76 (0.7)	1.53 (0.9)	0.032
**Losing confidence (−)**	1.63 (0.8)	1.45 (0.9)	n.s.
**Thinking of self as worthless (+)**	1.35 (0.8)	1.54 (0.9)	n.s.
**Feeling reasonably happy (+)**	1.06 (0.7)	0.92 (0.6)	0.098

**Table 6:** Distributions of the additional questions regarding health status (n.s. stands for not significant).

Items	Levels	Administrative/ Technical	Research/ Teaching	*p*-Value
**Psychological health**	Worsening	38 (33%)	94 (38%)	0.007
	Unchanged	43 (37%)	114 (46%)	
	Improvement	34 (30%)	38 (16%)	
**Anxiety**	Worsening	19 (20%)	74 (33%)	0.057
	Unchanged	13 (13%)	23 (10%)	
	Improvement	64 (67%)	128 (57%)	
**Demoralization**	Worsening	19 (20%)	70 (31%)	0.099
	Unchanged	16 (16%)	32 (14%)	
	Improvement	63 (64%)	124 (55%)	
**Items**	**Levels**	**Mandatory remote** **working**	**Voluntary remote** **working**	***p*-value**
**Psychological health**	Worsening	38 (33%)	24 (16%)	0.004
	Unchanged	43 (37%)	57 (39%)	
	Improvement	34 (30%)	65 (44%)	
**Anxiety**	Worsening	19 (20%)	25 (17%)	n.s.
	Unchanged	13 (13%)	19 (8%)	
	Improvement	64 (67%)	109 (75%)	
**Demoralization**	Worsening	19 (20%)	14 (9%)	0.058
	Unchanged	16 (16%)	20 (14%)	
	Improvement	63 (64%)	112 (77%)	

**Table 7:** GHQ distribution by gender in the three cohorts.

GHQ	Research/Teaching		Mandatory Remote Working (April 2022)		Voluntary Remote Working (December 2022)	
	*F*	*M*	*F*	*M*	*F*	*M*
**Typical**	16 (14%)	25 (19%)	27 (34%)	9 (24%)	42 (41%)	13 (30%)
**Evidence of distress**	65 (58%)	84 (63%)	36 (45%)	26 (68%)	50 (48%)	28 (65%)
**Severe problem**	32 (28%)	24 (18%)	17 (21%)	3 (8%)	11 (11%)	2 (5%)
***p*** **-value**	n.s.		0.047		n.s.	

**Table 8:** Differences between gender in relationship to task and type of remote working.

Item	Research/Teaching			Mandatory Remote Working			Voluntary Remote Working		
	*F*	*M*	*p*-Value	*F*	*M*	*p*-Value	*F*	*M*	*p*-Value
**GHQ total score (+)**	17.9	16.6	0.02	16.1	15.4	n.s.	15.0	15.6	n.s.
**Able to concentrate (+)**	1.23	1.16	n.s.	1.09	1.03	n.s.	1.00	0.98	n.s.
**Loss of sleep over worry (−)**	1.77	1.71	n.s.	1.58	1.58	n.s.	1.45	1.70	0.07
**Playing a useful part (+)**	1.19	1.10	n.s.	0.99	0.97	n.s.	0.87	0.95	n.s.
**Capable of makingdecisions (+)**	1.09	0.98	0.04	1.04	0.97	n.s.	0.96	0.95	n.s.
**Felt constantly under strain (−)**	2.25	2.04	0.02	1.92	2.06	n.s.	1.76	1.88	n.s.
**Couldn’t overcome difficulties (−)**	1.97	1.87	n.s.	1.92	1.83	n.s	1.53	1.65	n.s.
**Able to enjoy day-to-day activities (+)**	1.38	1.18	0.07	1.10	1.06	n.s.	1.06	0.95	n.s.
**Able to face problems (+)**	1.13	1.02	0.08	1.03	1.06	n.s.	1.01	0.84	0.06
**Feeling unhappy and depressed (−)**	1.83	1.86	n.s.	1.78	1.72	n.s.	1.47	1.67	n.s.
**Losing confidence (−)**	1.93	1.74	0.08	1.61	1.67	n.s.	1.37	1.63	0.08
**Thinking of self as worthless (+)**	1.12	1.27	n.s	1.34	1.36	n.s.	1.59	1.42	n.s.
**Feeling reasonably happy (+)**	1.27	1.08	0.02	1.13	0.92	n.s.	0.91	0.93	n.s.

## Data Availability

Data supporting the reported results can be found in the dataset stored in the electronic archives of the medical service, University of L’Aquila.

## Supplementary Materials

The following supporting information can be downloaded at: https://www.mdpi.com/article/10.3390/ijerph21040403/s1.
