# Supplementary material for: Study on Psychological Stress Perceived among Employees in an Italian University during Mandatory and Voluntary Remote Working during and after the COVID-19 Pandemic
Source: Int J Environ Res Public Health. 2024 Mar 26;21(4):403. doi: 10.3390/ijerph21040403 (PMC11050619; doi:10.3390/ijerph21040403)
Supplement: Supplementary file 1 [file ijerph-21-00403-s001.zip › ijerph-2849785-supplementary.pdf]

TELEWORKING IN THE UNIVERSITY

Study on Psychological Stress Perceived Among Employees in the University of L'Aquila during Mandatory and Voluntary Remote Working during and after the Covid-19 Pandemic

PART 1: INFORMED CONSENT AND DATA PROCESSING

INFORMATION SHEET AND INFORMED CONSENT

Dear Sir/Madam,
Our study aims to monitor the impact of smart working in terms of perceived productivity, personal and work well-being, and effects on psychological health among employees of the University of L'Aquila.

WHAT WILL MY PARTICIPATION IN THE STUDY MEAN?
If you choose to take part in the research, you will be requested to complete a questionnaire by providing answers to some questions.

WHAT ARE THE FORESEEABLE RISKS OF THE STUDY?
Participation in the survey does not entail any risk for you.

WHAT ARE THE EXPECTED BENEFITS OF THE STUDY?
The results of this study will contribute to deepening knowledge of the effects of smart working in terms of perceived productivity, personal and work well-being, and psychological health of employees who carry out their professional activities through this working method.

FREE CHOICE TO PARTICIPATE IN THE STUDY
The decision to participate in the study depends on you.
If you decide not to participate, this will not result in any modification to your usual professional activity, your working condition, or any other consequence.

CONFIDENTIALITY OF THE INFORMATION COLLECTED
If you decide to participate in the study, all the data collected will be processed and stored in a strictly anonymous manner pursuant to Legislative Decree 196/2003 and GDPR 2016 and subsequent amendments and additions regarding the processing of personal data. Only the personnel involved in the study will have access to this information.
Access to such data will be protected by the project supervisor, Prof. Leila Fabiani.
· I have read and understood this information sheet and its contents have been explained to me/us clearly and comprehensively.
· I understand that participation in the study is completely voluntary and that I may refuse to participate in the study
· Be aware that my clinical data will be made anonymous and may be used for scientific publications but will remain strictly confidential in compliance with current legislation and subsequent amendments and additions.

AUTHORIZATION TO PROCESS PERSONAL DATA

INFORMATION NOTE TO PARTICIPANT
PURSUANT TO THE LEGISLATIVE DECREE. 10 AUGUST 2018 N. 101 FOR ADAPTATION OF THE CODE REGARDING THE PROTECTION OF PERSONAL DATA (LEGISLATIVE DECREE 30 JUNE 2003 N. 196) AND TO THE PROVISIONS OF REGULATION (EU) 2016/679

Dear Sir/Madame,
We inform you that the processing of sensitive data collected during the study will be based on the principles of correctness, lawfulness, transparency, and confidentiality.

LEGAL BASIS AND PURPOSE OF THE PROCESS
Your data will be processed according to the responsibilities established by the rules of good clinical practice (Legislative Decree 211/2003) and the current legislation on the processing of personal data under Legislative Decree 10 August 2018 n. 101 of adaptation of the code regarding the protection of personal data (Legislative Decree 30 June 2003 n. 196) to the provisions
of Regulation (EU) 2016/679.
OWNER AND MANAGER FOR DATA PROCESSING AND PROTECTION
The data supervisor is Prof. Leila Fabiani, address: Occupational Medicine Service of the University of L'Aquila, room 12, Via G. Petrini, Rita Levi Montalcini building (Delta 6), 67100 Coppito, L'Aquila, Phone number. +390862434693, +393666214248 email: leila.fabiani@univaq.it.
Prof. Leila Fabiani, supervisor of the study, is also responsible for the processing, conservation, and protection of your data and can be contacted at the following addresses: Via G. Petrini, Rita Levi Montalcini building (Delta 6), 67100 Coppito, L'Aquila, Ph N. +390862434693, +393666214248 email: leila.fabiani@univaq.it.

The study's scientific staff includes:

Prof. Loreta Tobia Ph N +390862434645 email: loreta.tobia@univaq.it
Doctor Giada Mastrangeli Ph N +390862434695/96; email medicocompetente@strutture.univaq.it; Doctor Giulia Di Battista Ph N +390862434695/96 medicocompetente@strutture.univaq.it; Doctor Simona D'Onofrio simdonofrio@asl1abruzzo.it; Dr Pierpaolo Vittorini, email pierpaolo.vittorini@univaq.it; Prof. Leila Fabiani Ph N +390862434693; email leila.fabiani@univaq.it; Dr Pietro Di Benedetto email pietro.dibenedetto@univaq.it.

Methods for Data Treatment
Your data will be made anonymous and associated with a numerical code. They will be recorded, processed, and stored together with this code, kept in a password-protected folder on a password-protected PC located in a locked room at the University Occupational Medicine Service.
EXERCISE OF RIGHTS
At any time, you can exercise the rights referred to in the articles 7 of the Privacy Code and 15 of the European Regulation on the protection of personal data (e.g., access your data, integrate them, update them, correct them, oppose their processing for legitimate reasons, etc.) by contacting the Data Controller directly of the processing (Prof. Leila Fabiani Via G. Petrini, Rita Levi Montalcini building (Delta 6), 67100 Coppito, L'Aquila – phone number +390862434693, +393666214248 – email: leila.fabiani@univaq.it).

Part 2 – QUESTIONNAIRE

SOCIO-DEMOGRAPHIC AND EMPLOYMENT INFORMATION

Gender

- Female
- Male
- Unreported

Age group

- < 30
- 30-39
- 40-49
- 50-59
- >60

Where do you reside?

- Within the municipality of L'Aquila
- In a neighbouring municipality, still within the province of L'Aquila
- In another province

Normally, how long how long do you take to get to work??

- < 15 minutes
- 15 – 30 minutes
- 30 – 60 minutes
- >60 minutes

What vehicle do you use to get to work?

- Own means
- Public transport
- By foot
- Other

If you currently work from home (remote working), for how many hours?

- …hours
- I have used work from home in the past, not currently

If you currently work from home (remote working), on how many days?

- …days

Do you believe that the space and organization of the workstation where you work/have worked from home (good lighting, spacious work surface, ergonomic seat) are/have been:

- Satisfactory
- Indifferent
- Unsatisfactory

Do you believe that the supply of IT devices by the University for carrying out work from home (PC, printer, scanner...) is/has been:

- Satisfactory
- Indifferent

- Unsatisfactory

REMOTE WORKING EFFECTS ON WELL-BEING AND PSYCOLOGICAL HEALTH OF WORKERS

During the period of working from home the QUALITY of your night rest is:

- Increased
- Decreased
- Remained unchanged

During the period of working from home, the DURATION of your night rest is:

- Increased
- Decreased
- Remained unchanged

During the period of working from home, YOUR APPETITE is:

- Increased
- Decreased
- Remained unchanged

During the period of working from home, YOUR CONSUMPTION OF COMFORT FOODS (chocolate, ice cream, pizza, chips, biscuits...) is:

- Increased
- Decreased
- Remained unchanged

During the period of working from home (remote working), YOUR CONSUMPTION OF FRUIT AND VEGETABLES is:

- Increased
- Decreased
- Remained unchanged

During the period of working from home, were you able to KEEP PHYSICAL ACTIVITY?

- As usual
- Less than usual
- Not at all

During the period of working from home (remote working), HAS YOUR BODY WEIGHT CHANGED?

- yes, increased
- yes, decreased
- no, it has remained almost unchanged
- I don't know

During you’re the period of working from home, your ENERGY LEVELS:

- Increased, I felt more energetic
- Decreased, I felt more tired
- Remained mostly the same

During you’re the period of working from home, your CONCENTRATION ABILITY:

- Increased
- Decreased
- Remained mostly the same

During you’re the period of working from home, your PHYSICAL HEALTH:

- Improved
- Worsened
- Remained mostly the same

If you answered "Improved" or "Worsened" to the previous question, you believe this change was caused by:

- Remote working
- The lockdown and restrictions on movement and interpersonal contact due to the pandemic
- Both remote working and the lockdown
- Neither

During the period of working from home, overall, you believe that your PSYCHOLOGICAL HEALTH:

- Improved
- Worsened
- Remained mostly the same

If you answered "Improved" or "Worsened" to the previous question, do you believe this change was caused by:

- Remote working
- The lockdown and restrictions on movement and interpersonal contact due to the pandemic
- Both remote working and the lockdown
- Neither

During remote working, have you experienced any of the following symptoms?

	**Yes, I had never suffered from it in the past and I started**	**As usual, had already suffered from it before and it manifested itself with the same frequency and intensity**	**No, I did not suffer from it during the remote working period**
Heachache	◯	◯	◯
Gastritis	◯	◯	◯
Dermatitis	◯	◯	◯
Anxiety/restlessness	◯	◯	◯
Demoralization/sadness	◯	◯	◯
Back pain	◯	◯	◯
Muscle pain	◯	◯	◯

CURRENT WELL-BEING ASSESSMENT (GHQ-12)

The following questions ask about your CURRENT WELL-BEING.
Please answer the following questions based on the past 14 days.

In the last two weeks, have you been felt:

	**More than usual**	**As usual**	**Less than usual**	**Much less than usual**

able to concentrate on what you were doing (can you follow the thread of a conversation, concentrate at home and at work...)?	◯	◯	◯	◯
lose a lot of sleep, to the point where it's concerning?	◯	◯	◯	◯
productive in most of your activities?	◯	◯	◯	◯
able to make decisions in most cases?	◯	◯	◯	◯
constantly under pressure?	◯	◯	◯	◯
unable to deal with difficulties?	◯	◯	◯	◯
to find some time for yourself and enjoy it?	◯	◯	◯	◯
to deal with your problems?	◯	◯	◯	◯
unhappy or depressed?	◯	◯	◯	◯
like you've lost your confidence	◯	◯	◯	◯
like you have lower self-esteem?	◯	◯	◯	◯
in an overall happy emotional state?	◯	◯	◯	◯

REMOTE WORKING EXPERIENCE ASSESSMENT

Overall, do you think your remote working experience has been:

- Satisfying
- Neutral
- Unsatisfying

Which of the following statements do you most agree with?

- I would prefer a longer permitted remote working schedule
- The remote working contract is adequate
- I’m not interested in remote working, I prefer to work in presence

Do you think remote working affects your work productivity:

- For the better
- For the worse
- It’s indifferent

Do you think your ability to balance work and personal life during remote working has:

- Improved
- Worsened
- Remained the same

Do you think your relationship with your colleagues during remote working has:

- Improved
- Worsened
- Remained the same
